# Mindfulness Levels Among Oncology Nurses in Greece and Their Association With Demographic and Occupational Factors

**DOI:** 10.7759/cureus.111027

**Published:** 2026-06-17

**Authors:** Eleni Panagou, Stelios Parissopoulos, Theocharis I Konstantinidis, Ioanna Tsatsou, Ourania Govina

**Affiliations:** 1 Nursing Department, University of West Attica, Athens, GRC; 2 Department of Nursing, Hellenic Mediterranean University, Heraklion, GRC

**Keywords:** greece, health and well-being, mindful attention awareness scale (maas), mindfulness, oncology nurses

## Abstract

Introduction: Mindfulness is the continuous cultivation of awareness and attention to the present moment, which serves as a reference point for managing life situations. Its application in daily clinical practice improves the physical and psychological well-being of oncology nurses. The aim of this study was to assess the level of mindfulness among oncology nurses in Greece.

Methods: The study used a non-experimental, observational cross-sectional design with convenience sampling. It was conducted between March 2024 and December 2025 in eight tertiary hospitals in Attica, as well as in collaboration with the Hellenic National Nurses Association for hospitals outside Attica. A total of 200 nurses participated in the study. The Mindful Attention Awareness Scale (MAAS) was used and demonstrated excellent internal consistency, with a Cronbach’s alpha of 0.908.

Results: Eighty-nine-point five percent (89.5%) of the participants were women, with a mean age of 45 years and a high educational level (48.5% held an MSc or PhD degree). The participants demonstrated high levels of mindfulness, with a mean MAAS score of 63.55 (standard deviation (SD) = 12.60), which was significantly higher than the scale mean value (52.5). No statistically significant differences in the MAAS scores were observed in relation to the participants’ demographic and professional characteristics, including gender (*p = *0.518), marital status (*p =* 0.562), educational level (*p =* 0.617), job position (*p =* 0.296), employment sector (*p = *0.765), age (*p = *0.137), number of children (*p = *0.751), and years of oncology experience (*p = *0.770).

Conclusion: Oncology nurses in Greece demonstrated high levels of mindfulness, regardless of their demographic characteristics or work environment conditions.

## Introduction

Mindfulness refers to the continuous activation of the individual in a process of awareness and remembrance of the present as the only point of reference for managing any life situation. That is why the English term "mindfulness" has been attributed to the Greek language as "ensyneiditótita," since it seems to best represent the meaning that arises from the interpretation of the word. Thus, mindfulness forms a way of thinking, which is based on voluntary attention to the present moment, without criticism [1].

The concept of mindfulness refers to that state where the individual is fully aware of their feelings, thoughts, and overall experience, without attributing a negative or positive sign to them. Essentially, mindfulness demonstrates the transition from subjectivity to objectivity of the present and under the weight of only the circumstances of the moment [2]. Consequently, mindfulness refers to the non-self-critical acceptance of one’s feelings and thoughts, focusing one’s attention solely on the present. According to Grossman (2008), mindfulness is concerned with the terms of awareness, observation, and non-critical acceptance of thoughts and feelings [3]. In any case, the product of mindfulness is the result of reflection and calculation of present situations and does not arise as an automatic or automated reaction of the individual [4].

Mindfulness in recent decades seems to be gaining more and more ground in research conducted in the health sciences [5]. Undoubtedly, health professionals practice an extremely demanding profession, since they are faced with a multitude of incidents every day, many of which may be characterized as critical and urgent or on the verge of life and death. Thus, at a global level, burnout and work-related stress among healthcare workers are widespread [6]. These can be managed by implementing the practice of mindfulness, positively affecting the physical and mental health of health professionals [7].

Oncology nurses in their daily clinical practice face great and significant challenges (burnout, compassion fatigue, and moral distress). It is therefore important that they are able to maintain the appropriate balance in both their professional and personal lives and to enhance their physical, mental, and spiritual well-being, in order to continue to provide quality and compassionate care and for patients to express their satisfaction with it. Mindfulness is considered an effective method and practice with multiple benefits for the individual, such as stress and burnout reduction, mood improvement, and empathy cultivation, that could be utilized by oncology nurses [7,8].

The literature on mindfulness interventions specifically tailored for oncology nurses could not be described as particularly rich. Studies, such as that by Knill et al. (2021) concerning nurses working in hematopoietic cell transplantation [9], by Urso et al. (2022) on oncology intensive care unit (ICU) nurses [10], by Vaclavik et al. (2018) on nurses working in a hematology/oncology clinic [11], and by Qualls et al. (2022) concerning oncology nurses working with outpatients [12], are thus identified. These collectively underscore that mindfulness practices significantly mitigate professional burnout and compassion fatigue. These international investigations primarily focus on evaluating the efficacy of structured mindfulness-based stress reduction programs or brief digital interventions. The majority of existing evidence supports the positive effects of these interventions in enhancing psychological resilience, emotional regulation, and overall physical and psychological well-being among oncology nurses [8], which ultimately translates into safer, more compassionate patient care and improved clinical outcomes [13]. Therefore, the administrations of nursing institutions in general and nursing administrations in particular must invest in their staff and improve professional conditions, implementing interventions that will shield them against any adversities while maintaining their well-being and prosperity. In this context, improving the professional conditions and supporting nurses will improve the quality of their professional life [14]. Mindfulness practice is a relatively inexpensive and easy-to-implement intervention that can improve not only the professional quality of life of oncology nurses but also the outcomes for patients [8].

However, while international research heavily emphasizes the outcomes of active mindfulness interventions, there remains a paucity of baseline epidemiological data regarding the trait of mindfulness among oncology nurses within specific national healthcare structures. In Greece, despite the well-documented challenges of systemic understaffing and high clinical demands in oncology wards, relevant research activity remains critically limited. 

The present study addresses this distinct gap in the literature, offering the first research data on the evaluation of mindfulness levels among oncology nurses in Greece. By exploring how these scores interact with demographic and occupational factors, this research contributes novel insights into the structural and personal determinants of mindfulness. Ultimately, the primary goal of this research was to evaluate mindfulness levels among a sample of oncology nurses in Greece using the Mindful Attention Awareness Scale (MAAS) and to determine whether these levels vary significantly according to individual professional or demographic factors.

## Materials and methods

A cross-sectional study was conducted from March 2024 to December 2025 using convenience sampling in eight hospitals in Attica, in collaboration with the Oncology Nursing Sector of the Hellenic National Nurses Association (HNNA) for hospitals outside Attica.

Participant selection criteria were as follows: employment as clinical nurses in adult and pediatric oncology and/or hematology or surgical oncology departments, as well as palliative care units; clinical experience of at least one year; and the ability to speak and write in Greek. Resident nurses and those with a diagnosed psychiatric illness (depression, anxiety disorder, or substance dependence) were excluded. Ultimately, 200 nurses participated.

An a priori sample size calculation was not conducted due to the exploratory nature of the study, the baseline-mapping nature of this first-of-its-kind national study in Greece, and the use of convenience sampling. The study aimed to include the maximum feasible number of eligible oncology nurses during the data collection period. Although no formal sample size estimation was performed, the final sample of 200 participants was considered adequate for the planned statistical analyses and comparable to sample sizes reported in similar studies [8].

For the eight tertiary hospitals within the Attica region, the invitation link containing the study information and the electronic questionnaire was disseminated to the nursing staff via internal communication channels. For hospitals located outside the Attica region, recruitment was facilitated in collaboration with the Oncology Nursing Sector of the ΗΝΝΑ. The HNNA disseminated the study invitation electronically, embedding the survey link into their scheduled institutional emails and routine member communications.

At first, a self-designed, structured questionnaire was utilized to collect the participants' sociodemographic and professional background information. The demographic parameters included gender, age, marital status, and number of children. The occupational and work-related parameters evaluated were educational level, current employment department, current job position, work schedule, and total years of professional work experience specifically in the field of oncology.

The MAAS was used to assess mindfulness [15]. The MAAS is a 15-item unidimensional scale rated on a six-point Likert scale ranging from 1 (almost always) to 6 (almost never). It assesses mindfulness, defined as an individual’s general tendency to attend and to be aware of present-moment experiences in everyday life. Higher scores indicate higher levels of mindfulness. The scale focuses specifically on the presence or absence of attention and awareness in the present moment rather than on other mindfulness-related characteristics, such as acceptance, trust, gratitude, or non-judgmental attitudes. According to Brown and Ryan (2003), attention and awareness constitute the core structural components of mindfulness; therefore, other dimensions are not included in the instrument. Respondents are instructed to indicate how frequently they experience situations described in each statement based on what genuinely reflects their everyday experience rather than on how they believe they should behave. The items assess experiences of operating on “autopilot” or functioning without awareness of the present moment across cognitive, emotional, physical, interpersonal, and general domains [15].

The scale has demonstrated good psychometric properties and is considered suitable for adult populations regardless of meditation experience. It has also been associated with indicators of cognitive, emotional, functional, and physical well-being. Respondents rate how often they act “on autopilot” or operate without paying attention to the present moment. The Greek version of the MAAS was translated and validated by Mantzios et al. (2015) [16] and was used after permission from these authors. In the present study, the scale demonstrated excellent internal consistency (Cronbach's alpha = 0.908).

Ethics

The study protocol and the use of the questionnaire were approved by the Research Ethics Committee of the University of West Attica (reference number: 10597/20-02-2024). The questionnaires were distributed electronically to the target population. Participants were fully informed about the study and provided electronic informed consent prior to completing the questionnaires.

Statistical analysis

Prior to statistical analysis, the dataset was screened for completeness, normality, and potential outliers. No post-data-collection exclusions were performed beyond the predefined exclusion criteria. Cases with substantial missing data were not included in the final dataset; therefore, no imputation procedures were applied. Normality of continuous variables was assessed using the Kolmogorov-Smirnov test. Statistical outliers were examined descriptively, and no extreme values requiring removal or transformation were identified. No data transformations were performed, as the variables were considered appropriate for the planned analyses.

The values ​​of continuous variables are presented with mean and standard deviation or median. For categorical variables, frequencies (n) and percentages (%) were used. The analytic strategy was primarily exploratory and aimed to examine potential associations between MAAS and demographic or occupational characteristics. Group comparisons were performed using independent samples t tests, one-way analysis of variance, Mann-Whitney tests, or Kruskal-Wallis tests, depending on the normality of the data distribution. Correlation analyses were conducted using Pearson or Spearman correlation coefficients. The internal consistency reliability of the total score of the MAAS was estimated with the Cronbach's alpha coefficient.

To complement null-hypothesis significance testing, effect sizes were calculated using Cohen’s d for two-group comparisons (where values of 0.20, 0.50, and 0.80 represented small, medium, and large effects, respectively) and η2 for the multi-group comparison via ANOVA (where 0.01 represents a small effect).

Regarding data completeness and the management of missing data, the electronic questionnaire was structured using mandatory fields for all items. Consequently, participants could not submit the survey with incomplete data, ensuring a 100% completion rate for all submitted responses. Therefore, no statistical imputation procedures for missing values were required or applied to the final dataset of 200 participants.

All statistical analyses were performed with the statistical package IBM SPSS Statistics for Windows, Version 21.0 (released 2012, IBM Corp., Armonk, NY). All tests are two-sided. A p-value <0.05 was defined as a statistically significant difference, and marginal statistically significant differences (0.05 < p <0.1) will also be recorded.

## Results

Sample description

The study sample was mainly composed of women (n = 179, 89.5%). The average age of the participants was 45 years, while the majority were married or in a relationship (n = 134, 67%) with an average of two children. Regarding the educational level, the sample was characterized by high training, as almost half of the nurses (n = 97, 48.5%) had a master's or doctoral degree. Professionally, 55% (n = 110) worked exclusively in oncology departments, and 61% (n = 122) had the position of a clinical nurse, with the average experience in the field of oncology amounting to 9.5 years. In addition, 60.5% (n = 121) of the participants were employed under fixed working hours (Table 1).

**Table 1:** Demographic and work characteristics AEI: anotata ekpedeftika idrimata/universities, TEI: technologika ekpedeftika idrimata/technological educational institutes

Characteristic	Category	N	%
Gender	Male	21	10.5
	Female	179	89.5
Educational level	Bachelor's degree (TEI-AEI)	103	51.5
	Master's (MSc)	90	45.0
	Doctorate (PhD)	7	3.5
Marital status	Married/in a relationship	134	67.0
	Single	50	25.0
	Divorced	14	7.0
	Widowed	2	1.0
Department	Oncology	110	55.0
	Day clinic	72	36.0
	Other	18	9.0
Job position	Clinical nurse	122	61.0
	Nurse manager/supervisor	78	39.0
Work schedule	Fixed	121	60.5
	Rotating	79	39.5
Age (years)	Mean ± SD (min-max)	45.0 ± 8.9 (24-66)	
Number of children	Median (min-max)	2.0 (0-4)	
Work experience in oncology (years)	Median (min-max)	9.5 (1-37)	

Descriptive characteristics of MAAS

The assessment of mindfulness through the MAAS revealed a mean score of 63.55 (±12.60), with values ​​ranging from 27 to 86 (Table 2). This score is considered high, as the result is significantly above the theoretical average of the scale (52.5), suggesting that the participants have an increased ability to focus on the present and be aware while performing their tasks.

**Table 2:** Descriptive statistics of the MAAS MAAS: Mindful Attention Awareness Scale

Variable	Total score	Standard deviation (SD)	Minimum	Maximum	Scale midpoint (range)
MAAS	63.55	12.60	27.0	86.0	52.5 (15-90)

Association with demographic and occupational factors 

Subsequently, statistical analysis revealed that MAAS remained stable, regardless of the individual characteristics of the nurses (Table 3). In particular, no statistically significant differences were found in the MAAS in relation to gender (p = 0.518), marital status (p = 0.562), educational level (p = 0.617), or working hours (p = 0.518). Similarly, mindfulness did not appear to be affected by employment department (p = 0.765, η2 = 0.003) or position (p = 0.296, Cohen's d = -0.15), as clinical nurses and supervisors recorded similar performances. Finally, Pearson correlation analysis confirmed that factors, such as age (r = 0.105, p = 0.137), number of children (p = 0.751), and years of experience in oncology (p = 0.770), were not statistically related to the MAAS index (Table 4).

**Table 3:** Univariate analysis of MAAS in relation to demographic and work factors Note: d = Cohen’s d effect size for two-group comparisons; η2 = Eta squared effect size for multi-group comparison (ANOVA). MAAS: Mindful Attention Awareness Scale, ANOVA: analysis of variance

Characteristic	Category	Mean score	Standard deviation (SD)	p-value	Effect size
Gender	Male	65.24	15.33	0.518	d = 0.15
	Female	63.35	12.27		
Marital status	Married	63.19	12.58	0.562	d = -0.09
	Non-married	64.29	12.69		
Educational level	Bachelor's	63.12	12.64	0.617	d = -0.07
	MSc/PhD	64.01	12.60		
Department	Oncology	63.26	11.73	0.765	η^2^ = 0.003$
	Day clinic	63.47	13.92		
	Other	65.61	12.68		
Job position	Clinical nurse	62.80	12.82	0.296	d = -0.15
	Nurse manager/supervisor	64.72	12.23		
Work schedule	Fixed	63.59	12.64	0.518	d = 0.01
	Rotating	63.49	12.61		

**Table 4:** Correlation between MAAS and continuous demographic and work characteristics MAAS: Mindful Attention Awareness Scale

Characteristic	Correlation coefficient (r)	p-value
Age	0.105	0.137
Number of children	0.023	0.751
Work experience in Oncology	0.021	0.770

## Discussion

The demographic characteristics of the sample are consistent with the existing literature on nursing studies. Nursing is a predominantly female profession, with women comprising approximately 88-90% of the nursing workforce in the U.S. and around 77% globally. Also, most nurses worldwide belong to the age group of 35-44 [17].

The high educational level is also noteworthy, with 48.5% of participants holding a master's or doctoral degree. In Greece, only higher education institutions, specifically universities, provide nursing degrees. There are also a lot of opportunities for master’s and doctoral levels, similar to all university students. Master’s programs usually require tuition fees, while the PhD degree is offered at no cost. This is the reason behind this high educational level. Nevertheless, educational level or other personal or professional characteristics did not affect MAAS scores.

The participant nurses showed high levels of mindfulness, with a mean MAAS score of 63.55 ± 12.60. Similar findings were reported by the study of Fang et al. [18], where the MAAS score was 66.77 ± 10.41 in oncology nurses. Both studies report values ​​that are considered high and indicate an increased ability of oncology nurses to focus on the present during their work.

Furthermore, it is worth noting that, while in the international literature mindfulness is often examined as a regulatory factor of physical functions, such as in the study by Fang et al. [18], where a high score on the MAAS was associated with better sleep quality [18], the present research focuses on identifying the factors that shape mindfulness itself. Our findings highlight that mindfulness remains high and stable regardless of demographic characteristics, in contrast to other parameters of professional life (such as sleep) that seem to be influenced by marital status or previous employment [18].

In studies involving general nurses, similar high values appear. For example, clinical nurses in Beijing had an MAAS score of 66.82 ± 11.53. These scores were not related to demographics but to anxiety, stress, resilience, cognitive reappraisal, emotional exhaustion, and expressive suppression [19], whereas similar high values were obtained in nurses in Turkey [20].

Based on a recent systematic review, there is a wide variety of instruments to assess the mindfulness trait [8], but the MAAS was used in only one study that included oncology nurses [18]. This limits the possible discussion or comparison of our findings with other international studies. It is important to note that the researchers chose to use this tool because it has already been translated and validated in our native (Greek) language with good psychometric properties [16].

However, we have to note that despite the numerical convergence of high levels of MAAS scores across different research data, it is necessary to consider the role of the cultural context in interpreting these values. According to the recent review by Tsatsou [21], mindfulness is not a culturally neutral state, as the values, beliefs, and social norms of each country influence the way in which healthcare professionals perceive and express mindful attention. Cultural characteristics, such as collectivism versus individualism or the spiritual traditions of each society, shape the mindfulness trait in daily practice [21]. Therefore, while the high performance of oncology nurses in Greece on the MAAS is comparable to international data, it could tentatively be viewed in the light of the Greek culture of care and the particular characteristics of the domestic healthcare system, representing a plausible area for future empirical investigation.

Specifically for Greece, it can be hypothesized that the high level of mindfulness of oncology nurses may be linked to the conscious choice of engaging in a clinical field that requires constant alertness and mental availability. As Feng et al. reported [22], mindfulness in the nursing context is not simply a passive state, but a dynamic process of "conscious presence" and "conscious attention" that allows the nurse to respond effectively to clinical demands. The nature of oncology care in Greece, which is characterized by close interaction with the patient and their family environment, potentially serves as a contextual factor that may cultivate a form of “experiential mindfulness” [23].

According to Raingruber and Wolf [24], the nurse's ability to "be in the moment" is a central motivator and source of meaning in oncology practice, allowing the professional to identify care priorities within a high-stress environment. This skill functions as a protective mechanism, allowing the nurse to remain focused on the present and provide compassionate care, despite chronic pathologies and the shortcomings of the health system [24].

Furthermore, high mindfulness appears to enhance nurses’ professional autonomy, allowing them to make critical decisions and coordinate care with greater confidence. As Gagnon et al. [25] point out, autonomy in oncology care is inextricably linked to the nurse’s ability to respond mindfully to the complex needs of patients [25]. This ability not only improves nurse satisfaction but has a direct impact on patient safety and clinical outcomes, making mindfulness a building block of quality oncology care [26]. In conclusion, mindful presence emerges as a structural component of quality oncology nursing care, with a direct impact on nurses’ wellbeing [8, 22], patient safety, and clinical outcomes [26].

Limitations and strengths

The study focuses on a highly specialized and critical population, that of oncology nurses, who work under high-stress conditions. Despite the interesting findings, the present study is subject to certain limitations that must be taken into account when interpreting them. First, the cross-sectional design of the study does not allow for the investigation of causal relationships between variables, but only provides a snapshot of a given point in time. In addition, the use of convenience sampling may limit the generalizability of the results to all oncology nurses in the country, and more broadly, outside Greece and similar Mediterranean or resource-constrained healthcare systems. It must be pointed out that an a priori sample size calculation was not conducted due to the exploratory nature of the study. However, the approach of additional nurses through a scientific association (Hellenic National Nurses Association) significantly enhanced the geographical diversity and variation of the sample across Greece, capturing perspectives outside the capital city, even though it does not fully ensure statistical representativeness. A significant limitation concerns the heterogeneity of the sample in terms of gender, as the vast majority of participants were women, which makes statistical comparison between the two genders difficult. 

Furthermore, given the entirely electronic recruitment strategy, the study is susceptible to potential response and self-selection bias due to voluntary participation. It is plausible that oncology nurses who possessed a baseline interest in mindfulness, psychological well-being, or stress-reduction strategies were more motivated to complete the survey. This self-selection dynamic may have artificially skewed the findings toward higher MAAS scores, limiting the degree to which these results can be generalized to the broader oncology nursing workforce, who might be less engaged with such concepts. 

Another notable limitation is the lack of concurrent assessment of key psychological variables and potential confounders, such as professional burnout, work-related stress, anxiety, clinical resilience, or job satisfaction, which are heavily documented in international literature to interact with and influence mindfulness scores. Without controlling for these unmeasured psychological confounders, the interpretation of trait mindfulness remains strictly baseline and descriptive. Lastly, data collection relied on a self-report questionnaire (the MAAS), which may not only introduce social desirability bias but is also prone to potential ceiling effects, where participants might overestimate their present-moment awareness or cluster near the higher end of the instrument. 

Nevertheless, the scale is a weighted and reliable tool that presented excellent internal consistency, which in this particular study ensured the validity and reliability of the measurements. The high level of mindfulness recorded, combined with the fact that this skill appears independent of demographic and occupational limitations, effectively overcomes these constraints and offers a solid basis for planning future interventions in the field of employee mental health.

## Conclusions

The results of the present study highlight a particularly encouraging profile for oncology nurses in Greece, as the mean score on the MAAS indicates a high level of mindfulness and awareness in their daily practice. The fact that mindfulness appears independent of demographic factors, such as age and marital status, or professional variables, such as experience in oncology and type of schedule, suggests that mindfulness may operate as a robust, trait-like capacity among these professionals, appearing to remain consistently high despite the demanding and stressful conditions of the clinical environment. However, given the cross-sectional nature of this study, further longitudinal research is warranted to firmly establish the long-term stability of this skill. The lack of statistically significant differences between subgroups (e.g., between clinical nurses and supervisors) reinforces the view that mindful presence is a common characteristic of nursing staff in the oncology sector, regardless of hierarchical position or department of employment. Therefore, maintaining and further strengthening this skill through targeted interventions can act as a protective factor for nurses' mental resilience and improve the care provided to oncology patients.
